# A Defined Combination of Four Active Principles From the Danhong Injection Is Necessary and Sufficient to Accelerate EPC-Mediated Vascular Repair and Local Angiogenesis

**DOI:** 10.3389/fphar.2019.01080

**Published:** 2019-09-23

**Authors:** Shuang He, Hao Guo, Tiechan Zhao, Yanzhi Meng, Rongrong Chen, Jie Ren, Lanlan Pan, Guanwei Fan, Miaomiao Jiang, Gangjian Qin, Yan Zhu, Xiumei Gao

**Affiliations:** ^1^Tianjin State Key Laboratory of Modern Chinese Medicine, Tianjin University of Traditional Chinese Medicine, Tianjin, China; ^2^Research and Development Center of Traditional Chinese Medicine, Tianjin International Joint Academy of Biomedicine, Tianjin, China; ^3^Institute of Basic Medical Sciences, Xiyuan Hospital, China Academy of Chinese Medical Sciences, Beijing, China; ^4^Key Laboratory of Pharmacology of Traditional Chinese Medicine Formulae, Ministry of Education, Tianjin Key Laboratory of Traditional Chinese Medicine Pharmacology, and Institute of Traditional Chinese Medicine, Tianjin University of Traditional Chinese Medicine, Tianjin, China; ^5^Molecular Cardiology Program, Department of Biomedical Engineering, School of Medicine & School of Engineering, The University of Alabama at Birmingham (UAB), Birmingham, AL, United States

**Keywords:** ischemic vascular injury disease, Danhong injection, angiogenesis, EPCs, activity reconstitution

## Abstract

Many compounds in Chinese medicine formulae, including Danhong injection (DHI) formulae, are capable of stimulating angiogenesis and promoting vascular repair, but their chemical basis and action mechanisms remain poorly defined. The aim of this study is to determine the minimal native chemical composition of DHI for the pro-angiogenesis activity and to evaluate its contribution from local endothelial cells (ECs) and bone marrow-derived endothelial progenitor cells (EPCs). Our study demonstrated that the action of DHI in accelerating the recovery of hindlimb blood flow in a ischemic rat model was attributable to its local CXCR4-mediated pro-angiogenesis activity in mature endothelial cells, as well as to its ability to promote the proliferation, migration, adhesion, and angiogenesis of EPCs *via* integrated activation of SDF-1α/CXCR4, VEGF/KDR, and eNOS/MMP-9 signal pathways. Combination experiments narrowed down the angiogenic activity into a few components in DHI. Reconstitution experiment defined that a combination of tanshinol, protocatechuic aldehyde, salvianolic acid B, and salvianolic acid C in their native proportion was necessary and sufficient for DHI’s angiogenic activity. Compared with the full DHI, the minimal reconstituted four active principles had the same effects in promoting tube formation *in vitro*, improving perfusion and recovery of ischemic limb, and enhancing angiogenesis in ischemic mice post-hindlimb ischemia *in vivo*.

## Introduction

Cardiovascular and cerebrovascular ischemic diseases include a range of clinical syndromes induced by damage of vascular walls and are characterized by high morbidity, mortality, recurrence rate, and risk complications. These syndromes pose a serious health threat to humanity, especially to the elderly. Patients with cardiovascular and cerebrovascular diseases often require long-term medication for the treatment or prevention of recurrence. In severe cases, patients may even have to undergo surgery, and the resulting high medical costs produce a heavy financial and emotional burden on patients, especially those living in underdeveloped regions. Accordingly, the development of effective and inexpensive drugs for the prevention and treatment of cardiovascular and cerebrovascular diseases will deliver substantial economic and social benefits to China and the world as a whole. The herbal remedy is being incorporated into evidence-based medical practice as many phytochemicals have the effects of reducing the risk of various ailments (Al Disi et al., 2015). High blood pressure can be controlled or treated by lots of herbs (Anwar et al., 2016). The balance between herbal medicine and functional food can be used for the prevention and treatment of cardiac metabolism by modulating gut microbiota. (Lyu et al., 2017). Many kinds of plants show potentially beneficial effects to fight against arteriosclerosis by inhibiting the phenotypic switch of vascular smooth muscle cells (Saleh Al-Shehabi et al., 2016). Herbs play a role in improving the progression of CVD, particularly in terms of platelet function, and have the potential to alter platelet function tests and some coagulation parameters (McEwen, 2015).

Traditional Chinese medicine (TCM) is an ancient medical practice that has been clinically proven over thousands of years. It has made important contributions to health care in China and Asia. TCM is characterized by its simplicity, convenience, effectiveness, and affordability. The development of traditional Chinese medicine-based drugs is effective for prevention and treatment of cardiovascular and cerebrovascular diseases.

In TCM, great importance is attached to the normal circulation of *qi*, blood, and body fluid. Abnormalities of *qi*, blood, and body fluid will lead to diseases. Blood stasis is a common syndrome in the clinic and also is a shared syndrome of cardiovascular and cerebrovascular diseases. The principle of treating this condition is to promote blood circulation and remove blood stasis. Danhong injection (DHI) is a traditional Chinese medicine preparation composed of *Salvia Miltiorrhiza* and *safflower*. *Salvia Miltiorrhiza* promotes circulation and removes stasis while *safflower* stimulates blood circulation, regulates the meridians, eliminates stasis, and relieves pain. DHI has been widely used in Chinese hospitals and has been proven effective and safe for the treatment and prevention of various cardiovascular and cerebrovascular events, such as treating ischemia-reperfusion injury, atherosclerosis, acute coronary syndrome, and hepatic veno-occlusive disease (Yao et al., 2011; He et al., 2012; Liu et al., 2012). DHI can improve homocysteine, high-sensitivity C-reactive protein, and N-Terminal pro-brain natriuretic peptide to treat coronary heart disease unstable angina (Sun et al., 2014). The outcome of acute cerebral infarction in patients could significantly be improved by DHI after 14 days of treatment by reducing the inflammatory responses (Jiang and Lian, 2015). DHI can significantly improve the treatment of stroke and reduce the incidence of complications (Feng et al., 2019). Previous studies have shown that DHI could protect the integrity of the blood-brain barrier by promoting the growth of neural cells and endothelial cells, improve microcirculation, ischemia, and hypoxia in the brain, and prevent the development of cerebral vascular thrombosis by dilating cerebral vessels and reducing vasoconstriction (Y et al., 2006; F and CL, 2009). The above studies suggest that DHI affords unique advantages in the treatment of cardiovascular and cerebrovascular diseases (GY, 2012).

EA.hy926 is a cell line generated from the fusion of A549 cells and human umbilical vein endothelial cells. It is widely used as ECs model. Endothelial progenitor cells (EPCs) are endothelial-like cells isolated from human CD34+ cells *in vitro* by Asahara et al. (1997) in 1997. EPCs can act as endothelial cells and express endothelium-specific antigens. ECs and EPCs play an important role in the cardiovascular and cerebrovascular diseases, as they promote endothelial growth and neovascularization in the vascular injury parts. It confirmed that EPCs are involved not only in embryonic angiogenesis but also in angiogenesis postnatally (Murayama et al., 2002). About 25% of the ECs in new vessels are directly differentiated from EPCs (Suzuki et al., 2003). As the EA.hy926 and EPCs are indispensable in vascular repair, this study assessed the effects of DHI on their function. We chose some well-known genes in our study, such as growth factors, receptors, adhesion molecules, and enzymes for the gene regulatory network (Abdollahi et al., 2007; Seaman et al., 2007). These genes were supplied in the **Table S1**.

To determine the role DHI plays in vascular repair and find out the underlying mechanism DHI regulates, we established a hindlimb ischemia model in rats to examine the repair effects on the ischemic injury. We performed viability, migration, adhesion, and tube formation to assess the impacts of DHI on the function of EA.hy926 and EPCs, then proceeded to discuss the molecular mechanism by which DHI restored blood flow and improved ischemic injury. This study is to provide modern pharmacology-based experimental evidence for treating ischemic injury diseases and offering a scientific basis for the clinical application of blood circulation-promoting drugs.

DHI is widely used in the clinic, but the effective compounds of DHI are unclear. By integrating the data from our laboratory including chemical and pharmacological experiments of DHI, major compounds tanshinol (DSS), protocatechuic aldehyde (PAl), salvianolic acid B (Sal B), and salvianolic acid C (Sal C) were used to constitute reconstituted DHI according to the content that they were in DHI. To verify the pharmacological actions of reconstituted DHI, tube formation was detected by Operetta high content screening platform *in vitro*, and mice hindlimb ischemia model was used to verify the proangiogenic effects *in vivo*.

Exploring the pharmacodynamic substance basis is an important work in modern TCM. For a certain indication, not all components of the Chinese herbal compound are required. We choose the effective chemical composition to compose a preparation which has the same effect with DHI that will make the prescription more concise and the material foundation of efficacy clearer. Different combination of the chemical composition according to the indications can not only improve the efficacy but also make it more specificity.

## Materials and Methods

### Reagents

Dimethylbenzene, Heparin Sodium, ethyl alcohol, sodium chloride, potassium chloride muriate, disodium hydrogen phosphate, monopotassium phosphate, fibronectin (Fn) were purchased from Sigma Aldrich (Saint Louis, MO, USA). The von Willebrand factor (vWF, ab6994) was from Abcam (Cambridge, UK). Rhodamine-conjugated Goat anti-rabbit IgG polyclonal was from Jackson ImmunoResearch (Pennsylvania, USA). Bovine Albumin V (Beijing, China), DAPI was from Roche (Basel, Switzerland). We purchased the Basement Membrane Matrix (Matrigel^™^) from Becton Dickinson and Company (New Jersey, USA). Dulbecco Modified Eagle Medium (DMEM), Hoechst dye, fetal bovine serum (FBS), Calcein AM, and Trypsin-Ethylene Diamine Tetraacetic Acid (EDTA) (0.25%) were from Gibco (Invitrogen, California, USA). DMEM with high glucose and penicillin-streptomycin solution were from Hyclone (Logan, UT, USA). Human VEGF Immunoassay kit was from R&D Systems (Minnesota, USA). Cell Counting Kit-8 was from DOJINDO Laboratories (Kumamoto, Japan). Phosphate-buffered solution (PBS) was from Concord Technology (Tianjin, China). GenBond RNA extract Kit was from Renogen Biolab (Tianjin, China). Transcriptor First Strand cDNA Synthesis Kit was from Roche (Basel, Switzerland). Simvastatin tablets (H20080360) were purchased from Hangzhou MSD Pharmaceutical Co., Ltd. (Hangzhou, China). DHI, a traditional Chinese Materia Medica standardized product, was composed of extracts from Radix *Salviae miltiorrhizae* (Danshen) and *Flos Carthami tinctorii* (Honghua), and was supplied by Heze Buchang Pharmaceutical CO., Ltd. (China Food and Drug Administration Permission Number Z20026866). Sodium chloride injection (0.9%) was purchased from China Otsuka Pharmaceutical Co., Ltd. (Tianjin, China). For the animal study, low, medium, and high concentrations of DHI were prepared by freshly diluting with 0.9% sodium chloride (0.1, 0.3, 0.9 v/v). For cell culture study, DHI was diluted with DMEM medium by a factor of 10000, 3000, 1000, 300, and 100, respectively. Danshensu (DSS), salvianolic acid B (Sal B), and salvianolic acid C (Sal C) were obtained from Zhongxin Innova Laboratories (Tianjin, China). Protocatechuic aldehyde (PAl) was purchased from the National Institute for Food and Drug Control (Beijing, China). To determine the pharmacological action of the four active principles (DHI-C4), DSS (1.229 mg/ml), PAl (0.16 mg/ml), Sal B (0.595 mg/ml), and Sal C (0.00198 mg/ml) were diluted with 10% methanol according to their concentrations in DHI (Liu et al., 2013). For UPLC-UV analysis, 1-ml aliquots of DHI and DHI-C4 were diluted to 10 ml with 10% methanol aqueous solution.

### Animals

Wistar rats weighing 200 to 250 g and ICR mice weighing 20 to 25 g were purchased from Beijing HFK Bio-Science Company. We obtain the VEGFR-2-luc males (Ronicke et al., 1996; Zhang et al., 2004) from three transgenic breeding colonies maintained in the pathogen-free animal facility of Tianjin International Joint Academy of Biotechnology and Medicine (TJAB). The animals were housed in groups and maintained on a normal diet. All experimental protocols were reviewed and approved by the TJAB Animal Experimental Ethics Committee (TJAB-JY-2011-002) and conducted in accordance with the guidelines for animal experiments at Tianjin University of Traditional Chinese Medicine. Prior to the experiment, the animals were allowed to acclimate for 72 h at a constant temperature of 22°C in a 12-h light/dark cycled facility with free access to food and water.

### Rat Hindlimb Ischemia Model

Rats were anesthetized with 1.5% pentobarbital sodium (45 mg/kg), and unilateral hindlimb ischemia was induced as described previously (He et al., 2016). Briefly, two longitudinal incisions were made in the left thighs of the abdomen, parallel to the proximal arterial-venous-neural. The left external iliac artery and femoral artery were isolated from the vein and nerve; ligated twice, and then the artery was cut between the two ligatures. The right limb of each rat was used as an internal control. Laser Doppler perfusion imaging (LDPI) system (MoorLDLS, UK) was used to assess the limb perfusion noninvasively immediately after surgery and on postoperative days 3, 5, 7, 9, 12, and 14 at room temperature. The entire limb, including the foot, was gated to analyze the results. The average value of the blood perfusion of each limb was determined, and the ratio between the left and right limb was calculated. According to the weight and relative perfusion ratio, rats were randomly divided into five groups (n = 8 each) as follows: group 1 (control) was intravenously injected with 0.5-ml saline; group 2 (positive control) was intragastrically administrated with simvastatin (4 mg/kg); and groups 3, 4, and 5 were intravenously injected with different dose of DHI (0.25, 0.75, and 2.25 ml/kg), respectively.

### Capillary Count

Capillary density was measured by immunohistochemistry. On day 14, animals were sacriﬁced, and gastrocnemius muscle of both sides of hindlimbs was excised, ﬁxed immediately with 4% formalin for 48 h, and embedded in parafﬁn. Tissue blocks were cut into 3-μm-thick sections in sequential order transversely. Rabbit polyclonal anti-rat vWF antibody was used at 1:500 dilution at 4°C overnight, and a Rhodamine-conjugated Goat anti-rabbit IgG polyclonal was used as a secondary antibody at 1:1000 dilution at room temperature for 30 min. Images were captured by a ﬂuorescence microscope (200×), and the vWF-positive stain was counted as a capillary. Six random ﬁelds were chosen from different sections. Density was expressed as the mean number of capillaries per ﬁeld of view.

### Cell Viability Assay

EA.hy926 cells and EPCs were seeded into 96-well culture plates at a density of 5 × 10^3^ cell/well. After synchronization in serum-free DMEM for 12 h, the cells were incubated for 24 h in 1% FBS/DMEM with different dose of DHI (1:300, 1:1000, 1:3000, and 1:10000 dilution with DMEM medium). Cell viability was measured using Cell Counting Kit-8 (CCK8) following the manufacturer’s instructions using Microplate Reader (FlexStation^®^ 3, Molecular Devices, USA). Cell viability was expressed as the percentage of viable cells relative to untreated control cells using the absorbance at 570 nm.

### Cell Proliferation Assay

EPCs were seeded into 96-well culture plates at a density of 5 × 10^3^ cell/well. After cell synchronization in serum-free DMEM for 12 h, the cells were incubated for 48 h in 1% FBS/DMEM with different dose of DHI (1:300, 1:1000, 1:3000, and 1:10000 dilution with DMEM medium). Nucleus was labeled with 1 μg/ml Hoechst 33258 for 30 min in the dark and washed twice by PBS. Images were captured by Operetta High Content Analysis (HCA) System (PerkinElmer, Massachusetts, USA). Hoechst-positive cells were counted by Harmony High Content Imaging and Analysis Software. The ratio of cell numbers (drugs vs. control treatment group) reflects the proliferation ability.

### Cell Adhesion Assay

Cell adhesion assay was evaluated as described previously (Michaud et al., 2006). Cells were seeded into 6-well plates at a density of 5 × 10^5^ cells/well. Cells were treated with VEGF (50 ng/ml) or a different dose of DHI for 24 h, digested by pancreatic enzymes, collected and reseeded into 96-well culture plates at a density of 2 × 10^4^ cells/well. The medium was removed after 1 h incubation. Cells were fixed with 4% paraformaldehyde for 10 min, then 1 μg/ml Hoechst 33258 dye was added and incubated for 30 min in the dark, and washed twice by PBS. Nine random fields of view per well were examined. Cell images were captured by the Operetta HCA System, and the adhesion cells (Hoechst-positive) were determined by Harmony High Content Imaging and Analysis Software.

### Cell Migration Assay

EA.hy926 or EPCs were seeded into 96-well culture plates at a density of 2 × 10^4^ cells/well. After serum deprivation, cells were stained by 1 μg/ml Hoechst 33258 in the dark. Directional cell migration was analyzed by wound healing assay (Thompson et al., 2007). Scratches were made using a pipette tip, and cells were washed twice with PBS. The medium supplemented with VEGF (50 ng/ml) or different doses of DHI (1:3000, 1:1000, and 1:300 dilutions) were added into the wells separately, and plates were incubated at 95% air, 5% CO_2_, and 37°C. Cell migration was evaluated by measuring the distance between the scratch edges. Micrographs were obtained by the HCA System at 0 h, 4 h, 8 h, and 12 h. Image J Software was used to quantify the distance (pixel) of migration.

### *In Vitro* Tube Formation Assay

*In vitro* cell tube formation assay was performed following a procedure by Michaud (Michaud et al., 2003). The 96-well culture plates were coated with Basement Membrane Matrix. EPCs or EA.hy926 cells were incubated in medium with drugs at a density of 1.5 × 10^4^ cell/well. After 12 h incubation, cells were stained by Calcein AM for 30 min. Nine fields were randomly chosen in every well. Cell images were captured by Operetta HCA System. The ability of cell tube formation was analyzed by counting the number of tubes formed after drug treatment.

### Real-Time Quantitative PCR Analysis

The angiogenesis-related genes were examined by real-time quantitative PCR in ischemic gastrocnemius muscle or cultured EPCs treated with DHI. According to the manufacturer’s protocols, total RNA samples of the ischemic muscle or EPCs were isolated by using GenBond RNA extract Kit (Renogen Biolab, Tianjin, China). RNA samples were reverse-transcribed to complementary DNA (cDNA) by using Transcriptor First Strand cDNA Synthesis Kit (Roche, Switzerland) subsequently. cDNA was used as a template to do the real-time PCR ampliﬁcation. SYBR Green Master Mix reagent (Bio-Rad, 170-8880AP, USA) was used in qPCR to quantify the level of angiogenesis-related genes with B2M as an internal control. C1000™ Thermal Cycler Sequence Detection System (Bio-Rad, USA) was used to perform the amplification and analysis. After normalizing to a housekeeping gene using 2^−∆∆CT^, the fold increase or decrease was determined relative to the blank control.

### Quantitative Assay

UPLC-UV method was used to do the quantitative assay. We performed linearity, the limit of detection, the limit of quantiﬁcation, precision (intra-day and inter-day precisions), reproducibility, recovery, and stability tests in the methodological validation of the UPLC-UV method. A UV detector was used by setting detection wavelengths switching at different time intervals as follow: channel 1, 5.01 to 13.70 min, 286 nm for DSS and Pal; 14.50 to 45.50 min, 286 nm for Sal B and Sal C. During the analysis, the temperature of the sample manager was fixed at 4°C (Liu et al., 2013).

### Murine Hindlimb Ischemic Model

VEGFR-2-Luc mice were randomly divided into two groups (n = 3 each) according to body weight and relative perfusion ratio: groups 1 (control) and 2 (treatment) were intraperitoneally injected with 0.2-ml saline or DHI-C4 (equivalent to a human dose of 8.57 ml DHI), respectively. Mice were anesthetized with isoflurane and induced unilateral hindlimb ischemia as previously described (He et al., 2016). The entire femoral artery and vein of the left hindlimb were exposed, and the proximal and distal ends were ligated and excised in the middle. The right limb of each mouse was used as an internal control. Saline or DHI-C4 were administrated daily for 30 days after the surgery. LDPI system was used to assess the ischemic limb perfusion immediately after surgery and on postoperative days 0, 10, 14, 18, 21, 24, and 27. The ratio between the ischemic and non-operated limb (relative perfusion) was calculated.

### Bioluminescent Imaging *in Vivo*

We used IVIS Lumina K Series III system (PerkinElmer) to capture bioluminescence imaging (BLI) after HLI surgery and on postoperative days 6, 18, 21, and 30, with protocols similar to those described before (He et al., 2016). Mice were anesthetized with isoflurane and intraperitoneally injected with d-luciferin (PerkinElmer) at 150 mg/kg according to the manufacturer recommendations. The optical signal intensity of the VEGFR-2-Luc mouse was acquired 5 min after D-luciferin administration. We identified the regions of interest (ROI) from displayed images on the ischemic sites by using Living Image1 software (He et al., 2016).

### Micro-CT Scanning

*In vivo* angiogenesis was determined by micro-CT scanning as previously. In brief, after exposure to saline or DHI-C4 for 30 days, the mice were anesthetized and injected with Omnipaque (20 ml/kg) intra-arterially at 1.6 ml/min, and the hindlimbs were scanned by micro-CT (Quantum FX, PerkinElmer) for 5 min, and angiogenesis was measured with computer software (Quantum FX UCT system).

### Statistical Analysis

Data analyses were performed with Origin 8.5.1 software, and the signiﬁcant difference was analyzed by SPSS16.0 statistical software. The results were shown as mean ± SD. Statistical significance was computed by unpaired Student’s *t*-test for observation between 2 groups or by analysis of variance test for comparison between multiple groups. *P < 0.05 was considered to be statistically significant.

## Results

### DHI Improved Recovery of Ischemic Limb Perfusion in Rats

**Figure 1:**
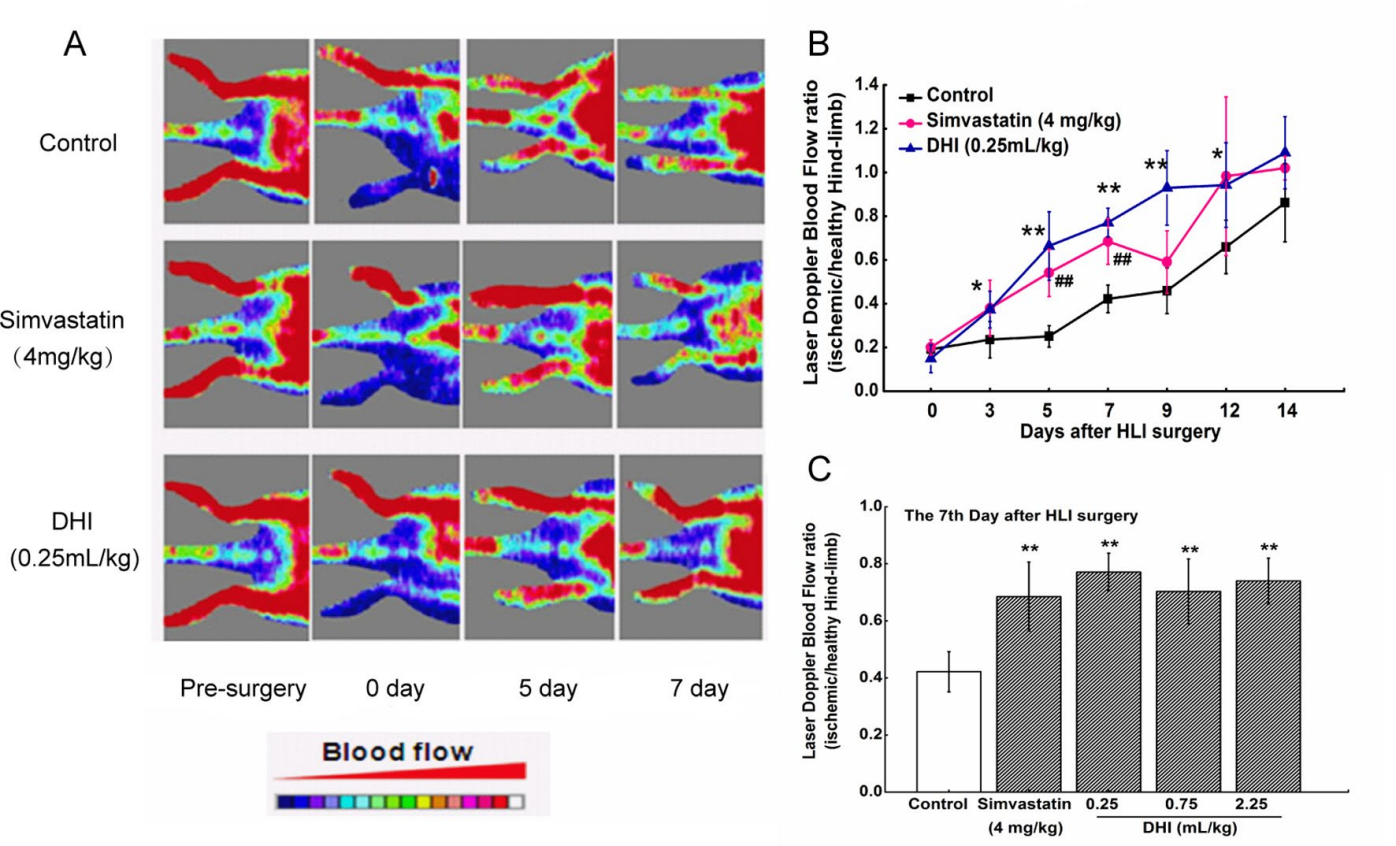
DHI improved perfusion of ischemic limbs in ischemic rats. **(A)** Representative images of laser Doppler perfusion analysis for the control group, simvastatin group, and DHI (0.25 ml/kg) group before surgery and at different time points after surgery. Rats were administered with drugs for 14 consecutive days starting on the first day after surgery. Blood flow was recorded by laser Doppler flowmeter at day 0, 3, 5, 7, 9, 12, and 14 after surgery. The shift from blue to red in color indicates an increase in blood flow. **(B)** The mean hindlimb blood flow was calculated as the ratio of ischemic (left) side to non-ischemic (right) side. DHI (0.25 ml/kg) or simvastatin significantly improved perfusion recovery after HLI surgery. **(C)** All concentrations of DHI improved perfusion recovery in the ischemic rat model on the seventh day after surgery. *P < 0.05, **P < 0.01 DHI groups compared with the control group, ^##^ *P < 0.01*, simvastatin group compared with the control group.

LDPI was performed before, immediately after, and over 14 days after hindlimb ischemia surgery to evaluate the effect of DHI on blood perfusion. In saline-treated rats, blood flow recovered to a ratio of 0.42 ± 0.06 after 7 days, whereas in DHI-treated rats (0.25 ml/kg), the LDPI ratio was accelerated to 0.77 ± 0.07 after 7 days (**Figure 1**). In addition, at day 7 after surgery, low, medium, and high concentrations of DHI- (0.25, 0.75, and 2.25 ml/kg) or simvastatin-treated rats showed a significantly better recovery of the limb perfusion (**Figure 1C**). Therefore, blood flow recovery in an ischemic rat model was severely impaired, and DHI or simvastatin could improve the deficiency significantly.

### DHI Increased the Capillary Density and Increased the Expression of CXCR4 in Hindlimb Gastrocnemius of the Ischemic Rat

**Figure 2:**
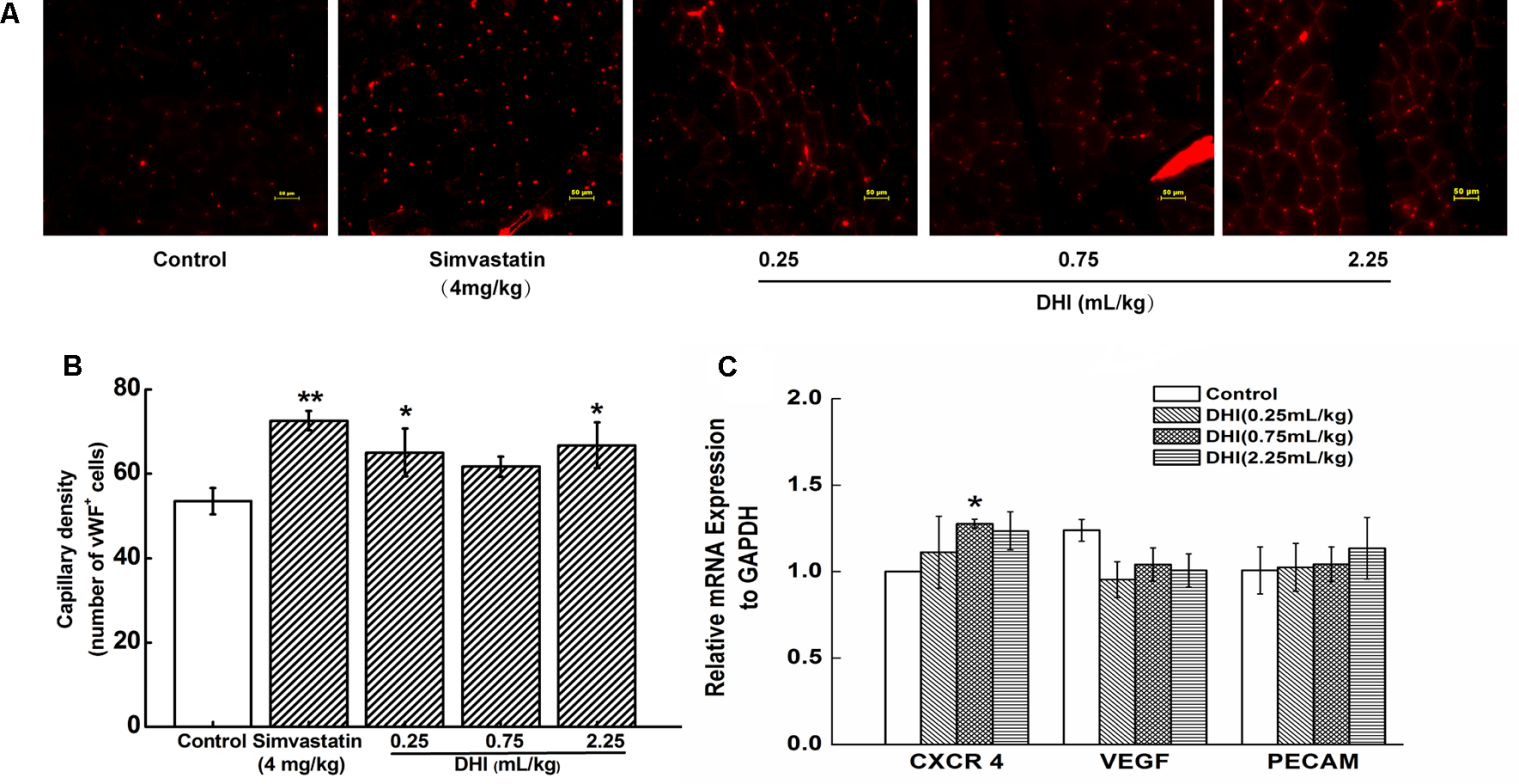
DHI increased capillary density post HLI and increased CXCR4 expression in ischemic muscle tissue. **(A)** Representative immunofluorescent images of ischemic limb muscle 2 weeks after treatment. Five visual fields were randomly chosen from each section. A vWF-positive stain was counted as a capillary. **(B)** Quantitative analysis of the capillary density of the ischemic region was performed at the end of week 2 for ischemic rat model. *P < 0.05, **P < 0.01, compared with the control group (n = 3). **(C)** Quantitative PCR showed DHI increased the expression of CXCR4 in ischemic gastrocnemius muscle. *P < 0.05 vs. control group. Data represent the mean ± SD. The experiments were performed in duplicate and confirmed the reproducibility.

Immunohistochemical staining for endothelial-specific vWF was used to quantify the capillary density in tissue sections. We found that the total number of capillaries in the tissue sections of simvastatin-treated group (4 mg/kg) was significantly higher than that of the control group (72.60 ± 2.26 vs. 53.53 ± 3.11, **P < 0.01). Similarly, low and high concentrations of DHI (0.25 and 2.25 ml/kg) significantly increased the capillary density in the hindlimb gastrocnemius of the ischemic rat (65.04 ± 5.71, 66.71 ± 5.41 vs. 53.53 ± 3.11, compared to the control group, *P < 0.05). The medium concentration of DHI also led to an increase in the capillary density, but not to a significant level (**Figures 2A, B**). These results provided evidence that DHI treatment promoted restoration of blood flow partially by increasing capillary density in the ischemic area. Increased CXCR4 expression in the ischemic gastrocnemius muscle of DHI-treated mice was detected, whereas those of VEGF and PECAM were not changed (**Figure 2C**).

### DHI Improved Endothelial Cells Function

**Figure 3:**
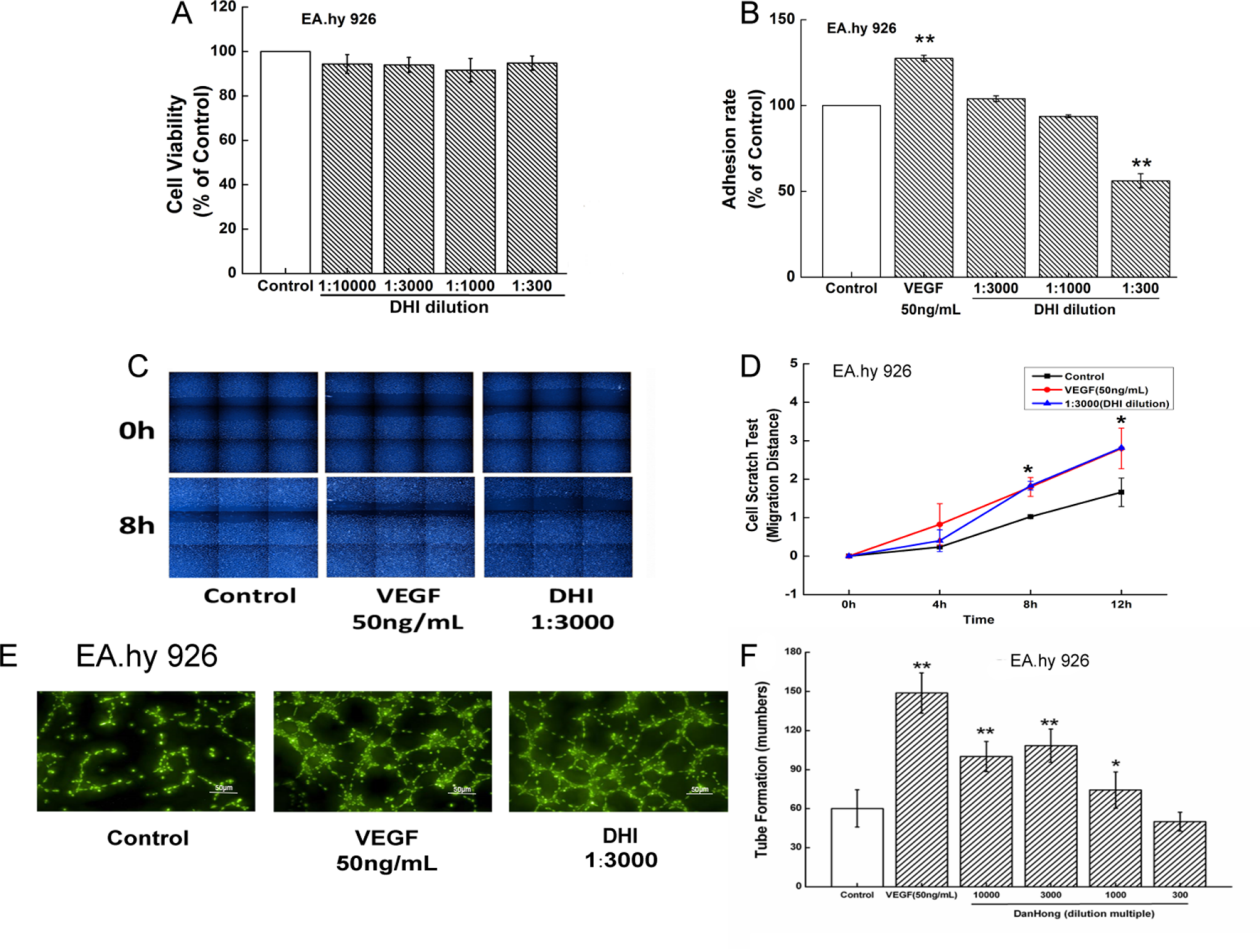
DHI improved endothelial cells function. **(A)** EA.hy926 viability was determined by Cell Counting Kit-8 assay under different concentrations of DHI, none dilution caused any significant viability changes. **(B)** VEGF (50 ng/ml) significantly increased adhesion ability. Lower concentrations (3000, 1000-fold dilution) of DHI did not change the adhesion ability. However, higher concentration (300-fold dilution) of DHI decreased the hy926 adhesion ability compared with the control group. **(C)** Representative images of the wound healing assay in EA.hy926 cells. **(D)** Quantitative analysis of the migration distance, VEGF or DHI (3000-fold dilution) promote the cell migration after 8 h. **(E)** Microscopic images showing tube formation of EA.hy926 cells. **(F)** After incubating for 12 h with DHI, different dilution folds of DHI promoted angiogenesis in EA.hy926 cells. Data represent the mean ± SD. *P < 0.05, **P < 0.01, compared with the control group.

To determine the safe range of DHI in *in vitro* cell experiments, cell viability tests were performed. Incubation of EA.hy926 cells with DHI diluted up to 300-fold did not cause any significant viability changes (**Figure 3A**). To determine if DHI affects the adhesion of mature endothelial cells, *in vitro* cell adhesion assay were performed using EA.hy926 cells. Compared with the control group, VEGF (50 ng/ml) significantly increased adhesion ability. Lower concentration of DHI (3000- and 1000-fold dilution) did not change the adhesion ability. However, a higher concentration of DHI (300-fold dilution) decreased adhesion ability (**Figure 3B**). VEGF (50 ng/ml) and DHI (3000-fold dilution) significantly increased migration rate compared with the control group (**Figures 3C, D**). In an *in vitro* angiogenesis assay, VEGF (50 ng/ml), DHI diluted 3000, 1000, and 300 folds all promoted tube formation ability compared with the control group (**Figures 3E, F**), confirming the proangiogenic capability of DHI in mature vascular endothelial cells.

### DHI Improved EPCs Function

**Figure 4:**
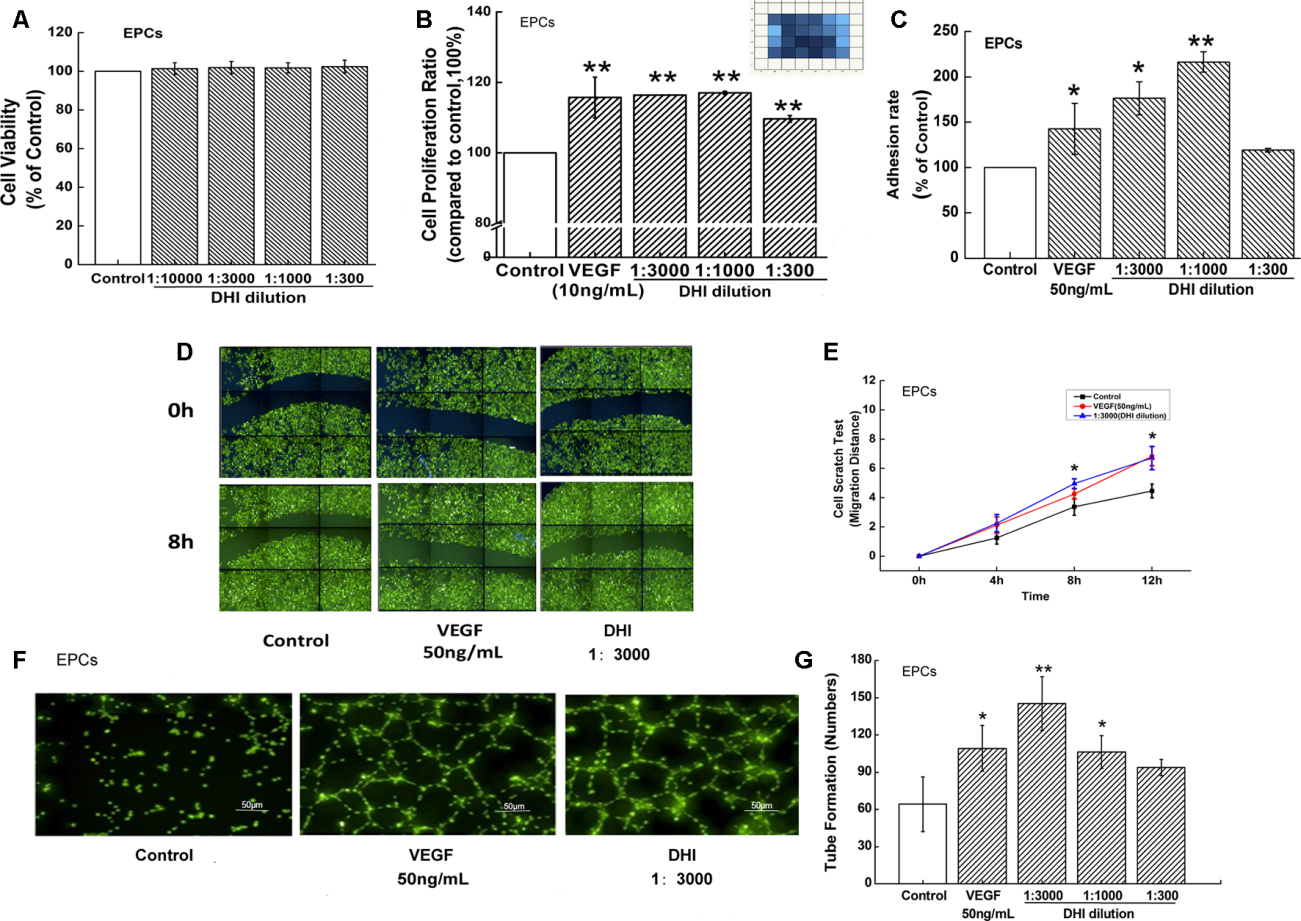
DHI improved EPCs function. **(A)** EPCs viability was determined by Cell Counting Kit-8 assay under different concentrations of DHI, none dilution caused any significant viability changes. **(B)** Different concentrations of DHI significantly increased EPCs proliferation after 48-h cultivation. **(C)** VEGF (50 ng/ml) and lower concentrations of DHI (3000- and 1000-fold dilution) increased the adhesion ability significantly whereas the highest concentration of DHI (300-fold dilution) had no effect on EPCs adhesion ability. **(D)** Representative images of the wound healing assay in EPCs. **(E)** Quantitative analysis of the migration distance, VEGF or DHI (3000-fold dilution) promoted the cell migration after 8 h. **(F)** Microscopic image showing tube formation of EPCs. **(G)** After incubating for 12 h with DHI, difference dilution folds of DHI promoted angiogenesis in EPCs. Data represent the mean ± SD. *P < 0.05, **P < 0.01, compared with the control group.

To examine the role of DHI on EPCs, a cell viability test was first performed. Incubation of EPCs with DHI in the range from 10000- to 300-fold dilutions did not affect EPC viability (**Figure 4A**). The effect of DHI on the proliferation of human EPCs was then examined. Similar to the positive control VEGF (10 ng/ml), different concentrations of DHI (3000-, 1000-, and 300-fold dilutions) significantly increased EPCs proliferation after 48 h cultivation (**Figure 4B**). We used CCK8 for the viability assay, Operetta HCA System for the proliferation assay. We think that by measuring a population of cells, CCK8 is a less sensitive assay compared to HCA, which counts individual cells. In addition, the duration of drug treatment was different in the two experiments. These may be the reasons why there be no change in viability but an increase in proliferation. *In vitro*, EPC cell adhesion assays showed that lower concentration of DHI (3000- and 1000-fold dilutions) increased the adhesion ability significantly whereas a high concentration of DHI (300-fold dilution) did not (**Figure 4C**). VEGF (50 ng/ml) and DHI (3000-fold dilution) significantly increased migration rate compared to the control group (**Figures 4D, E**). Finally, both VEGF (50 ng/ml) and DHI (3000- and 1000-fold dilutions) promoted tube formation compared with the control group (**Figures 4F, G**). Taken together, these results suggested that DHI is capable of promoting angiogenesis by promoting proliferation, adhesion, and migration in EPCs.

### Effects of DHI on the Expression of Angiogenesis-Related Genes in EPCs

**Figure 5:**
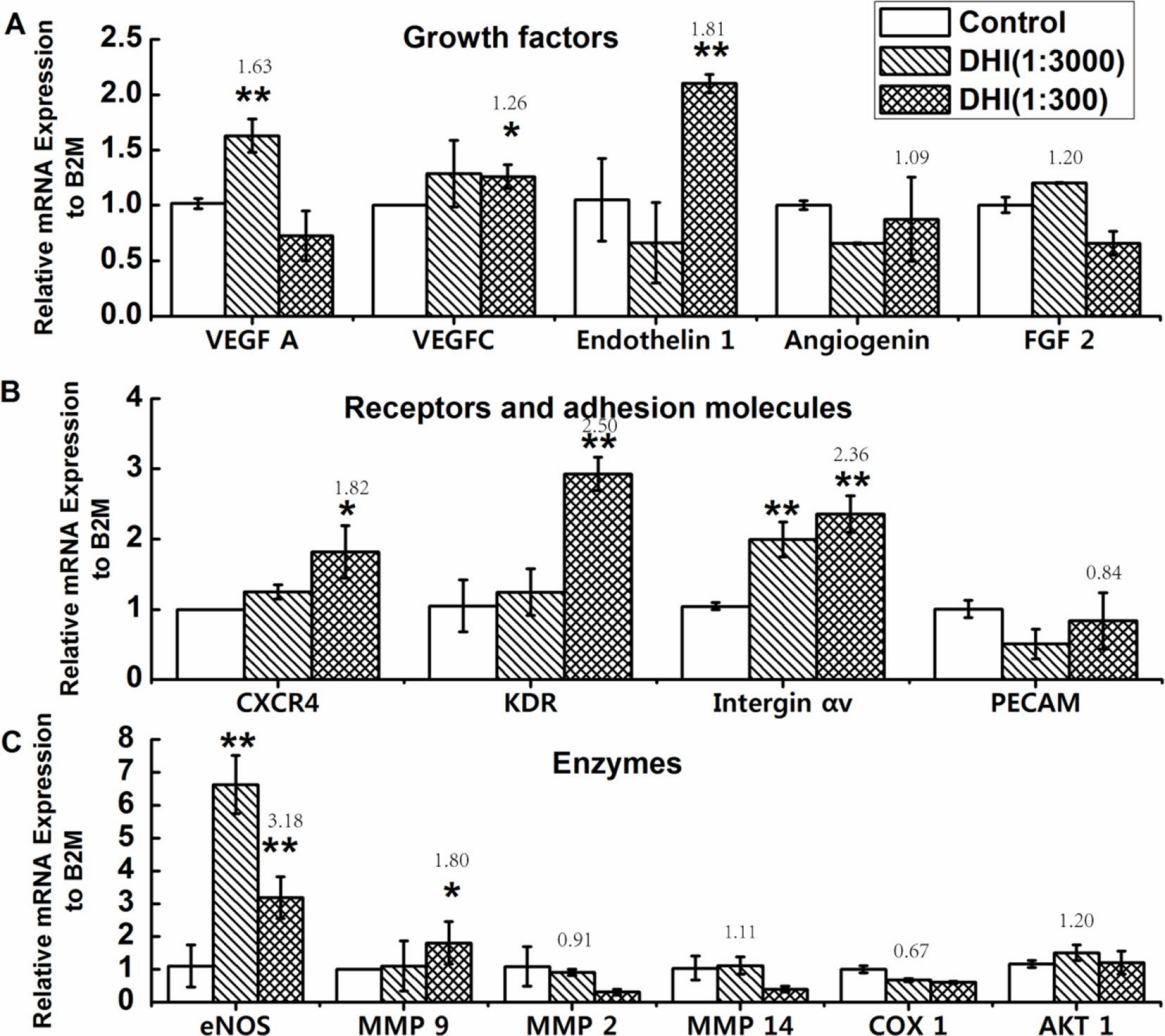
Effects of DHI on the expression of angiogenesis related genes in EPCs. EPCs were treated with DHI (3000 and 300, dilution folds) for 24 h, then qPCR assay was performed. **(A)** Quantitative PCR showing increased expression of VEGFA, VEGFC, and Endothelin 1 in EPCs treated with DHI. **(B)** Quantitative PCR showing increased expression of CXCR4, KDR, and Integrin αv in EPCs treated with DHI. **(C)** Quantitative PCR showing increased expression of eNOS and MMP9 in EPCs treated with DHI. *P < 0.05, **P < 0.01 vs. control group. Data represent the mean ± SD. All experiments were performed in duplicate and confirmed the reproducibility.

Quantitative PCR (qPCR) analyses were carried out to access the influence of DHI on the regulation of angiogenesis pathways in EPCs. As shown in **Figure 5**, DHI promoted the expression of multiple angiogenesis-related genes, including growth factors (VEGFA, VEGFC, and Endothelin 1), receptors (CXCR4 and KDR), adhesion molecules (Integrin αv), and enzymes (eNOS and MMP-9), while it had no effect on others, such as Angiogenin, FGF-2, PECAM, MMP-2, MMP-14, COX-1, and AKT-1.

### Defining and Reconstituting the Chemical Composition of Pro-Angiogenesis Activity of DHI

**Figure 6:**
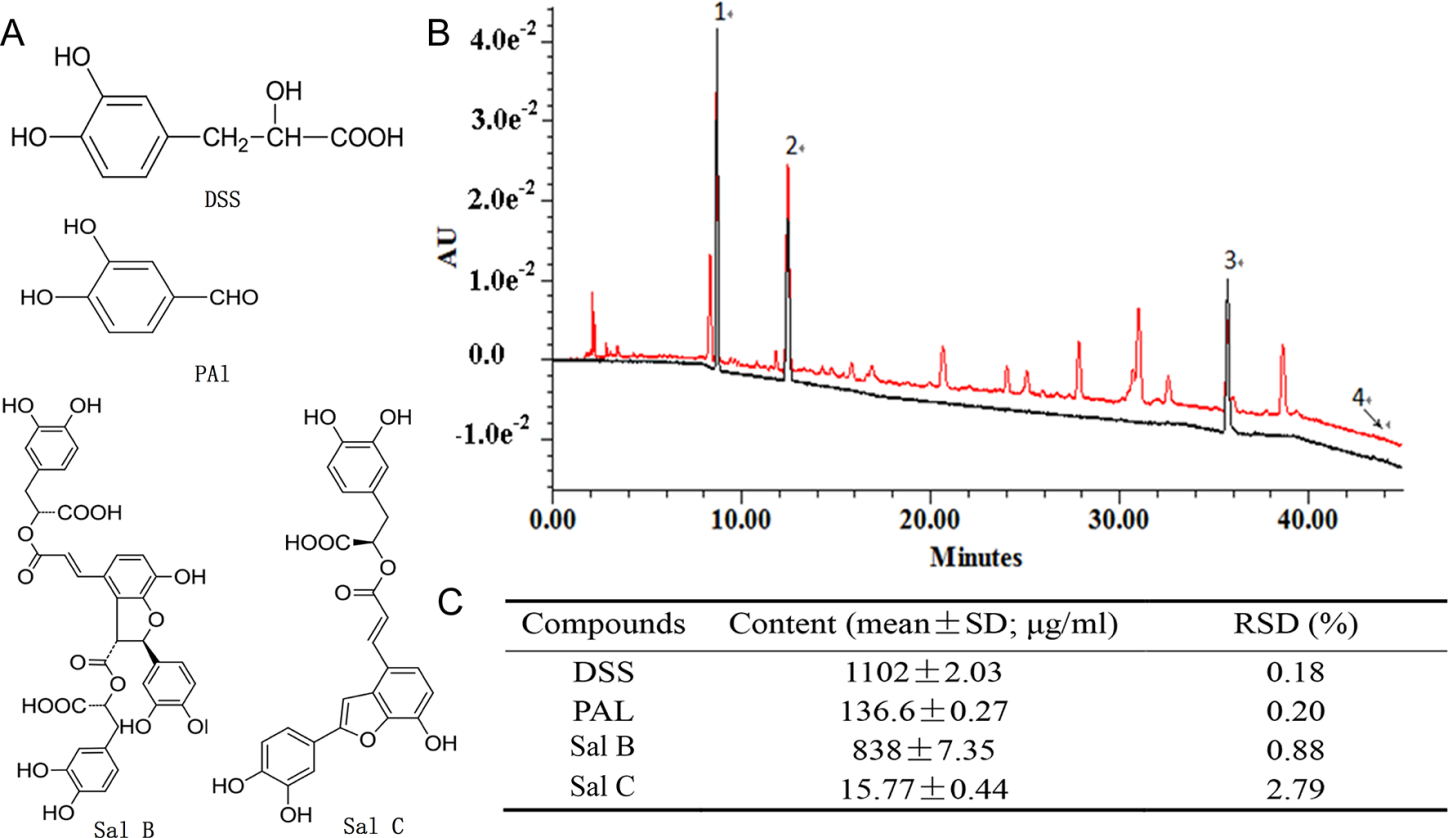
Minimal reconstituted DHI with four compounds. **(A)** Chemical structures of DSS, Pal, Sal B, and Sal C. **(B)** UPLC-UV chromatograms of DHI (black) an/d DHI-C4 (red). Peaks: (1) tanshinol (DSS), (2) protocatechuic aldehyde (PAl), (3) salvianolic acid B (Sal B), and (4) salvianolic acid C (Sal C). **(C)** Quantitation of the four compounds in DHI-C4.

To identify the chemical basis of DHI in promoting EPC-dependent angiogenesis, two different activity-based screen approaches were taken. First, as we reported earlier (Zhao et al., 2017), a chemical fraction library of DHI was constructed and screened by *in vitro* tube formation assay. Fractions with higher angiogenesis activities were identified, but they represented only partial activity compared with DHI (data not shown). A reconstitution approach was then taken by combining the nine major constituents in DHI (Liu et al., 2013) in exactly the same proportion as in DHI and testing its pro-angiogenesis activity. Compared with DHI, this “reconstituted” DHI with nine major components (DHI-C9) retained a similar pro-angiogenesis activity by the tube formation assay (data not shown). Screening different combinations of DHI-C9 in tube formation assay defined a minimal pro-angiogenesis activity consists of DSS, PAl, Sal B, and Sal C (DHI-C4). As shown in **Figure 6**, the UPLC chromatographic profile of DHI-C4 was identical to that of DHI.

### DHI-C4 Promoted Tube Formation *in Vitro* in Both Endothelial Cells and EPCs

**Figure 7:**
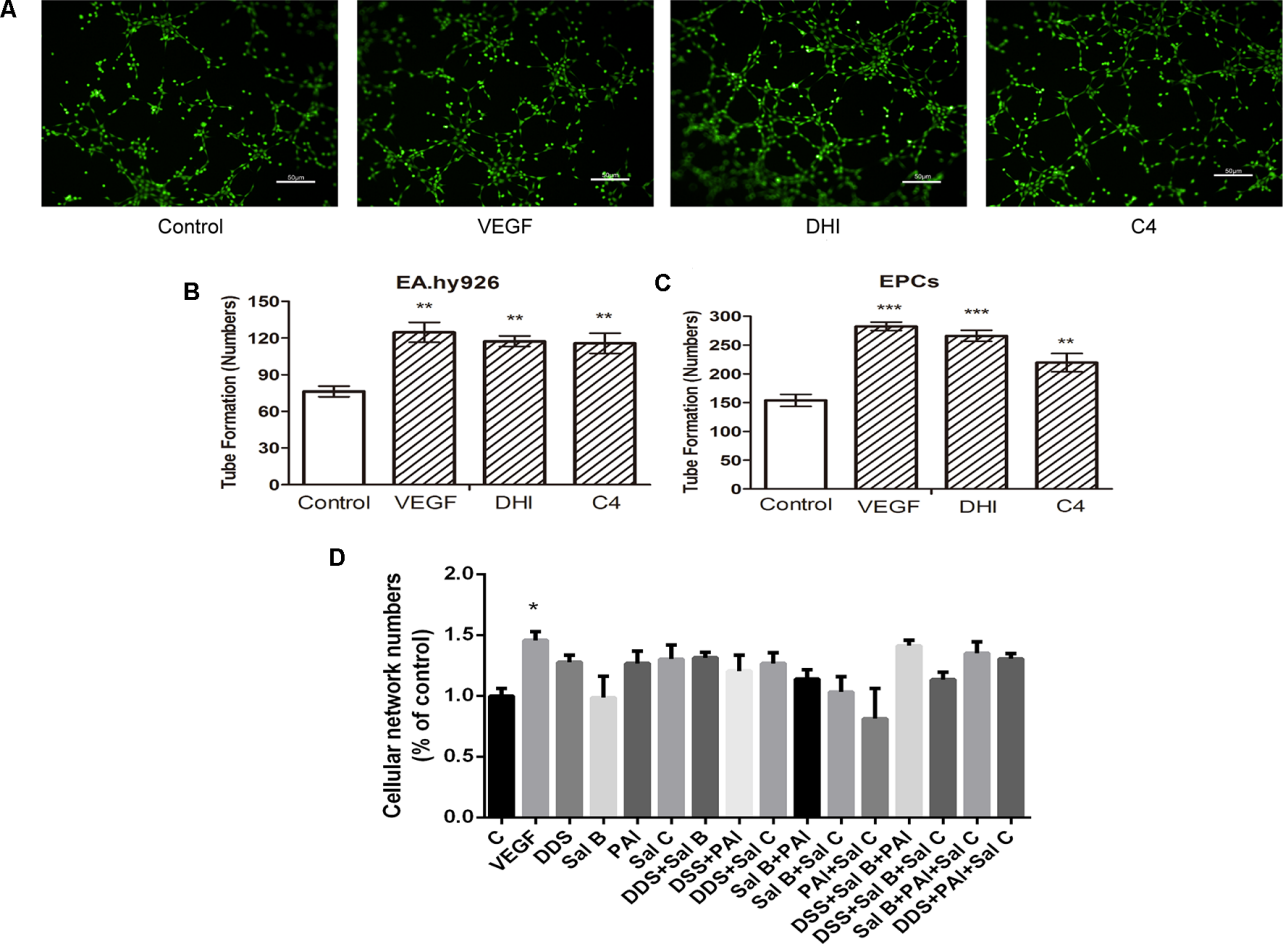
DHI-C4 promoted tube formation in both endothelial cells and EPCs. **(A)** Microscopic image showing tube formation of EA.hy926 cells. **(B)** VEGF, DHI, and DHI-C4 promoted angiogenesis, respectively, in EA.hy926 cells. **(C)** VEGF, DHI, and DHI-C4 promoted angiogenesis, respectively, in EPCs. Data represent the mean ± SD. **P < 0.01, ***P < 0.001, compared with the control group. **(D)** Each component of DHI-C4 respectively or two components of DHI-C4 or three components of DHI-C4 did not promote tube formation in endothelial cells compared with the control group. The data are a representation of the mean ± SD. *P < 0.05, compared with the control group.

The pro-angiogenesis activity of DHI-C4 was verified in both EA.hy926 cells and EPCs (**Figure 7A**). The results showed that DHI-C4 had the same effect as VEGF and DHI to promote tube formation compared with the control group in both cells (**Figures 7B, C**). These results confirmed that the four components from DHI were sufficient to promote angiogenesis in EA.hy926 and EPCs. Tube formation experiment was then performed with each individual component of DHI-C4 as well as the combinations of two or three components. The results (**Figure 7D**) showed that any reduction from DHI-C4 would significantly diminish its pro-angiogenesis activity, suggesting that the four components from DHI-C4 were necessary to promote angiogenesis in endothelial cells.

### DHI-C4 Improved Recovery of Ischemic Limb Perfusion and Enhanced Angiogenesis *in Vivo* in Ischemic Mice

**Figure 8:**
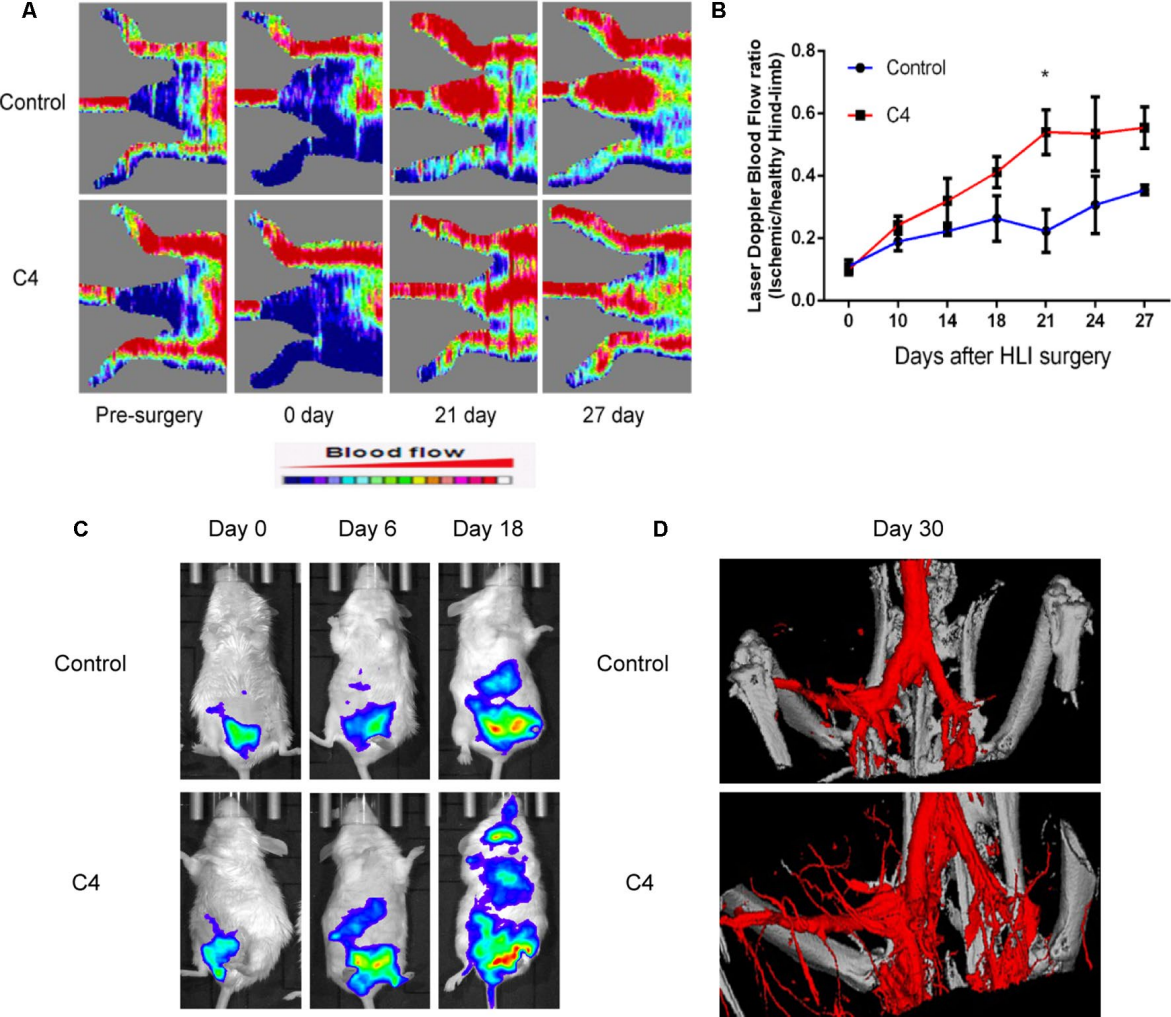
DHI-C4 improved perfusion of ischemic limbs and enhanced local angiogenesis in ischemic mice *in vivo*. **(A)** Representative images of laser Doppler perfusion analysis for control mice and mice treated with DHI-C4 before surgery and at different time points after surgery (n = 3). **(B)** The mean hindlimb blood flow was calculated as the ratio of ischemic side to non-ischemic side. DHI-C4 significantly improved perfusion recovery after HLI surgery at 21 days. *P < 0.05, DHI-C4 group compared with the control group. **(C)** Representative bioluminescent images of VEGFR-2-Luc mice after HLI were obtained at 18, 21, and 27 days under the same imaging conditions. **(D)** Representative images of micro-CT reconstructed 3D microangiography of the defect areas (n = 3).

To evaluate the effect of DHI-C4 on blood perfusion in ischemic mice, we used LDPI to monitor hindlimb blood flow before surgery and during 27 days after surgery. The ratio of blood flow between the two hindlimbs was about 1.0 before the operation, and the ratio of blood flow between the ischemic and non-ischemic limbs was 0.104 ± 0.03 after surgery. Blood flow recovered to a ratio of 0.36 ± 0.02 in saline-treated mice after 27 days, whereas the LDPI ratio was accelerated to 0.55 ± 0.13 in DHI-C4-treated mice after 27 days. In particular, DHI-C4-treated mice showed significantly better limb perfusion recovery on day 21 (**Figures 8A, B**). The effect of DHI-C4 on ischemia-induced angiogenesis could be evaluated by the IVIS system, as angiogenesis biomarker (VEGFR-2) could be visualized directly through bioluminescent imaging *in vivo*. There was no difference in average bioluminescent intensities between control and DHI-C4-treated group before and on day 6 after HLI surgery, DHI-C4 treatment led to a significant increase in bioluminescent intensity at the ischemic area at 18 days (**Figure 8C**), suggesting an upregulation of local VEGFR-2 expression coincident with accelerated blood-flow perfusion recovery. After 30 days, the mice were scanned by micro-CT to evaluate the new microvasculature formation in the HLI area. As shown in **Figure 8D**, a much larger vascular network was generated in DHI-C4-treated mice compared to that of the control group.

## Discussion

The pathology of many ischemic cardiovascular and cerebrovascular diseases is related to the damage of vascular walls. They are usually manifested as blood stasis syndrome in the practice of traditional Chinese medicine. DHI was formulated based on the TCM theory of promoting blood circulation and removing blood stasis (PBCRBS). Our previous study shows that DHI effectively dissolves thrombus and ameliorates its derived dry gangrene (Zhao et al., 2017). Yang et al. recently showed that DHI reduces vascular remodeling and up-regulates the Kallikrein-Kinin system in spontaneously hypertensive rats (Yang et al., 2017). Therefore, there is a great prospect of DHI in treating cardiovascular and cerebrovascular diseases.

Angiogenesis is the process through which new blood vessels are formed in the existing vascular bed in a sprouting or non-sprouting manner through endothelial cell proliferation and migration. It is closely related to many physiological and pathological processes (Hughes et al., 2006; Werner and Nickenig, 2006) and plays an important role in improving repair outcome after tissue ischemia. Using different rodent models, we and Wu *et al*. have shown that DHI effectively increases blood flow recovery after tissue ischemia in diabetic mice by promoting angiogenesis (Wu et al., 2015; He et al., 2016). In the present study, we further demonstrated the contribution of EPCs by showing that DHI could significantly enhance the proliferation, migration, adhesion, and formation of a tube-like structure in EPCs. We used CCK8 for the viability assay, while cell proliferation was examined by counting the number of stained nuclei using HCA System. We think that by measuring a population of cells, CCK8 is a less sensitive assay compared to HCA assay, which counts individual cells. In addition, the duration of drug treatment was different in the two experiments. Cells were treated with the drug for 24 h in the viability assay, whereas they were treated with the drug for 48 h in the proliferation assay. These may be the reasons why there be no change in viability but an increase in proliferation.

Furthermore, our study explored that DHI up-regulated the VEGF-A, VEGF-C, and VEGFR2 in cultured EPCs. VEGF is an important growth factor family, it can specifically bind to its receptor (VEGFR) to stimulate the proliferation and differentiation of vascular endothelial cells, promote the formation of collateral vessels, and ensure the establishment of microcirculation during ischemic tissue recovery (Hariawala et al., 1996; Jiang et al., 2008).

SDF-1 is involved in tissue regeneration by significantly promoting the mobilization of bone marrow-derived cells to the myocardial ischemic area (Askari et al., 2003). CXCR4 is the only receptor for SDF-1 and is a G protein-coupled receptor, playing an important role in enhancing the angiogenesis of EPCs *in vivo* (Chen et al., 2010). The interaction between SDF-1 and CXCR4 is of great significance for a number of cell functions including migration, mobilization, and homing (Yamaguchi et al., 2003). The present study found that DHI could significantly up-regulate the expression of CXCR4 in ischemia gastrocnemius muscle and cultured EPCs. The results indicate that SDF-1/CXCR4 may be one of the molecular mechanisms by which DHI promoted angiogenesis by improving the function of EPCs.

The adhesion ability of ECs and EPCs is important to angiogenesis when EPCs mobilized and homed to the ischemic area. Our results showed that DHI promoted the expression of integrin αv which is related to the positive effect on the adhesion function of EPCs. eNOS is a key enzyme in maintaining the endothelial functional integrality. Meanwhile, it is also a downstream pathway, by which some cytokines, including VEGF, ANG-1, and so on, promote the proliferation, mobilization, migration, and angiogenesis in EPCs (Duda et al., 2004). Matrix metalloproteinase 9 (MMP-9) plays a key role in mobilizing bone marrow-derived EPCs to the ischemic area. When NO or SDF-1 enter the bone marrow microenvironment, they will induce the activation of MMP-9 and release the soluble kit-ligand (sKitL). Subsequently, sKitL induces the release of more SDF-1, enhancing mobilization of EPCs to the circulation (Petit et al., 2007). We found that DHI can increase gene expression of eNOS and MMP-9. This indicates that eNOS/MMP-9 activation may be one of the molecular mechanisms by which DHI induces angiogenesis.

It is well known that the complexity of ingredients restricts the development of TCM. Although there are many pro-angiogenesis Chinese medicine formulas that have been used extensively in clinics, the efficacy of these formulas is demonstrated by the whole prescription and their effective components are not clear. DHI-C4 is derived from a TCM, DHI, which has been widely used in the clinic for treating cardiovascular diseases. We selected the major active components DSS, PAl, Sal B, and Sal C according to the content ratio in DHI. Our study is the ﬁrst to use a group of chemicals according to the natural proportion of a complex Chinese medicine formula. As the main component of DHI-C4, DSS has been reported to accelerate angiogenesis after myocardial infarction in rats (Yin et al., 2017). Protocatechualdehyde has shown vascular protective potency (Kong et al., 2016). Other studies also found that Salvianolic acid can exert cardioprotection through promoting angiogenesis in animal models of myocardial infarction. Sal B was reported to enhance angiogenesis (Lay et al., 2003) and exerts beneficial cardioprotective effects on acute myocardial infarction by promoting neovascularization (Lin et al., 2016). Transplantation of Sal B pretreated mesenchymal stem cells improves cardiac function in rats with myocardial infarction through angiogenesis (Guo et al., 2014a). We demonstrated that a combination of DSS, PAl, Sal B, and Sal C, as well as DHI, can synergistically promote angiogenesis. It is the first time to restructure the active principles from the effective TCM to restore the efficacy of the whole prescription. HY926 cells and hindlimb ischemia model have been widely used for studies of ischemic cardiovascular diseases. Our findings facilitate an understanding of the detailed mechanisms by DHI-C4 in vascular repair. Yu and coworkers explored a combination of four active compounds alleviates cerebral ischemia–reperfusion injury in correlation with inhibition of autophagy and modulation of AMPK/mTOR and JNK pathways, while the four compounds combined in a certain ratio (6:9:5:4) they selected according to the optimization condition in animal models not the natural proportion of them in Sheng-mai San (Guo et al., 2014b). However, the mode of action and related signaling pathways of DHI-C4 are unknown. Further experiments are needed to test the mechanism of the compounds and their mixtures to clarify the interaction between the compounds.

Hindlimb ischemia model is a well-established model for therapeutic angiogenesis studies (Limbourg et al., 2009). However, the proper use of this model and interpretation of the data are necessary (Limbourg et al., 2009). We have previously used this model to show that DHI effectively promoted blood flow recovery in diabetic mice (He et al., 2016). It is worth noticing that the blood flow recovery was much slower in diabetic mice (up to 35 days, He et al., 2016) than in normal mice (∼18 days, **Figure 8**). In either case, the *in vivo* angiogenic-promoting gene (such as VEGFR2) expression correlated nicely with the recovery of the local blood flow in the HLI model. However, over a long period of time past recovery phase, VEGFR2 expression may fluctuate due to factors unrelated to the angiogenic process (data not shown).

## Conclusion

In this study, we investigated the role of DHI in the repair of ischemic vascular damage at the molecular, cellular, and animal levels. Numerous results showed that DHI could integrate the activation of SDF-1α/CXCR4, VEGF/KDR, and eNOS/MMP-9 signal pathways together, thereby promoting the proliferation, migration, adhesion, and angiogenesis of ECs and EPCs. This may be the mechanism by which DHI increased capillary density and promoted blood flow in the ischemic area, suggesting that DHI plays an important role in repairing the vascular injury. Reconstituted DHI which including DSS, PAl, Sal B, and Sal C had the same effects to promote tube formation *in vitro* and improve recovery of ischemic limb perfusion by enhancing angiogenesis in HLI ischemia mice *in vivo*. Therefore, a defined combination of four active principles from DHI is necessary and sufficient to accelerate vascular repair, and this discovery provides better guidance for a new direction for further development of natural combination medicine.

## Data Availability

All datasets generated for this study are included in the manuscript/**Supplementary Files**.

## Ethics Statement

This study was carried out in accordance with the recommendations of Committee of Ethics on Animal Experiments at the Tianjin International Joint Academy of Biomedicine. The protocol was approved by the Tianjin International Joint Academy of Biomedicine.

## Author Contributions

YZ conceived and organized the study. HG, SH and RC performed the experiments to evaluate the pro-angiogenesis activity of DHI and prepared **Figures 1**–**5**. YM and LP performed the quantitative assay and tube formation assay of reconstituted DHI and drew **Figures 6**–**7**. TZ and JR performed the *in vivo* experiment of reconstituted DHI and drew **Figure 8**. GF, MJ and GQ helped with the design of the study and interpretation of results. HG, SH and YZ wrote the manuscript. XG and YZ reviewed and edited the final manuscript.

## Funding

This study was financed in part by the grants from National Science Foundation of China (81873037, 81503292), Major National Science and Technology Projects (2018YFC1704500) and the outstanding youth fund project for the independent theme of Chinese Academy of traditional Chinese medicine (ZZ0908021).

## Conflict of Interest Statement

The authors declare that the research was conducted in the absence of any commercial or financial relationships that could be construed as a potential conflict of interest.
