# Supplementary material for: A Defined Combination of Four Active Principles From the Danhong Injection Is Necessary and Sufficient to Accelerate EPC-Mediated Vascular Repair and Local Angiogenesis
Source: Front Pharmacol. 2019 Sep 23;10:1080. doi: 10.3389/fphar.2019.01080 (PMC6767990; doi:10.3389/fphar.2019.01080)
Supplement: Supplementary file 1 [file Table_1.docx]

Table 1S

| Name | Gene Bank Accession# | | | Primer Sequence | Position | | | | Length (bp) | |
| --- | --- | --- | --- | --- | --- | --- | --- | --- | --- | --- |
| B_2_M | NM_004048.2 | | F:TGCTGTCTCCATGTTTGATGTATCT | | 674-  759 | | | | | 86 |
|  |  |  | R:TCTCTGCTCCCCACCTCTAAGT | |  |  |  |  |  |  |
| CXCR4 | NM_003467.2 | | F:CTTCAGTTTGTTGGCTGCGG | | 97-213 | | | | | 117 |
|  |  |  | R:AGTGTATATACTGATCCCCTCCA | |  |  |  |  |  |  |
| VEGF C | NM_005429.2 | F:CTTCCTGCCGATGCATGTCT | | | | 1126 -1221 | | | | 96 |
|  |  | R:CTGCCTGACACTGTGGTAGT | | | |  |  |  |  |  |
| Integrin αv | NM_002210.3 | | F:CCGCGCCTTCAACCTAGAC | | 2302-  378 | | | | | 142 |
|  |  |  | R:AAGAAACATCCGGGAAGACAC | |  |  |  |  |  |  |
| HIF-1α | NM_001530.3 | | | F:GAGGGAGCCAGCGCTTAG | 233-  358 | | | | | 116 |
|  |  |  |  | R:ACTTATCTTTTTCTTGTCGTTCGC |  |  |  |  |  |  |
| AKT1 | NM_001014432.1 | | | F:GCACAAACGAGGGGAGTACA | 369-  479 | | | | | 111 |
|  |  |  |  | R:TCACGTTGGTCCCACTCCTG |  |  |  |  |  |  |
| integrinβ3 | NM_000212.2 | | | F:AAGGATAACTGTGCCCCAGA  R:CACAGGCTTGTCCACAAATG | 174-  569 | | | | | 396 |
| KDR | NM_002253.2 | | | F:CACCACTCAAACGCTGACATGTA  R:GCTCGTTGGCGCACTCTT | 1616-  1710 | | | | | 94 |
| FGF-2 | NM_002006.4 | | | F:GTGTGTGCTAACCGTTACCT  R:GCTCTTAGCAGACATTGGAAG | 696-  932 | | | | | 237 |
| PECAM1 | NM_000442.4 | F:TCTATGACCTCGCCCTCCACAAA  R:GAACGGTGTCTTCAGGTTGGTATTTCA | | | | | | 2953-  3035 | | 83 |
| MMP-2 | NM_004530.4 | | | F:GCGGCGGTCACAGCTACTT  R:CACGCTCTTCAGACTTTGGTTCT | 2185-  2255 | | | | | 71 |
| MMP-9 | NM_004994.2 | | | F:TGGGGGGCAACTCGGC  R:GGAATGATCTAAGCCCAG | 1032-  1111 | | | | | 80 |
| MMP14 | NM_004995.2 | | | F:ACATTGGAGGAGACACCCAC  R:TAGGCAGTGTTGATGGACGC | | | 857-  1349 | | | 493 |
| VEGF-A | NM_001025366.2 | | | F:AGGGCAGAATCATCACGAAGT  R:AGGGTCTCGATTGGATGGCA | | | 1137-  1121 | | | 75 |
| ANG | NM_001097577.2 | | | F:CTGGGCGTTTTGTTGTTGGTC  R:GGTTTGGCATCATAGTGCTGG | 133-  245 | | | | | 113 |
| EDN1 | NM_021729.4 | | | F:TCCTGCTCTTCCCTGATGGA  R:TGCTCAGGAGTGTTGACCCA | 491-  567 | | | | | 77 |
| Cox-1 | YP_003024028.1 | | | F:CGATGCATACACCACATGAA  R:AGCGAAGGCTTCTCAAATCA | 5904-  7445 | | | | | 100 |
| eNOS | NM_000603.4 | | | F:ctcatgggcacggtgatg  R:accacgtcatactcatccatacac | 1821-  2005 | | | | | 184 |

Table S1. Primers used in the quantitative reverse transcription–polymerase chain reaction (qRT-PCR).
